# Respiratory symptoms, vaccination coverage, and antibiotic use among Hajj pilgrims: a multi-season cross sectional study (2023–2025)

**DOI:** 10.3389/fpubh.2026.1885706

**Published:** 2026-07-10

**Authors:** Fadi S. I. Qashqari

**Affiliations:** Department of Microbiology and Parasitology, College of Medicine, Umm Al-Qura University, Makkah, Saudi Arabia

**Keywords:** antibiotic use, antimicrobial stewardship, Hajj, mass gathering medicine, mass gatherings, respiratory tract infections, vaccination, vaccine coverage

## Abstract

**Background:**

Respiratory tract infections are the most frequently reported illnesses during Hajj, one of the world’s largest recurring mass gatherings. The convergence of millions of pilgrims from diverse geographic regions in Makkah, Saudi Arabia creates conditions conducive to the transmission of respiratory pathogens. Understanding patterns of infection, vaccination coverage, and antibiotic use across multiple seasons is essential to inform preventive strategies.

**Methods:**

A multi-season cross-sectional study was conducted during three consecutive Hajj seasons (2023–2025). Adult pilgrims were recruited and interviewed using a standardized structured questionnaire. Data collected included sociodemographic characteristics, vaccination history (meningococcal, influenza, COVID-19, pneumococcal), comorbidities, smoking behavior, respiratory symptoms, healthcare-seeking behavior, and antibiotic use. Multivariable logistic regression analyses were performed to identify factors independently associated with respiratory symptoms and antibiotic consumption.

**Results:**

A total of 712 human participants (adult Hajj pilgrims) were enrolled. Overall, 38.9% reported at least one respiratory symptom at the time of assessment, with cough (28.1%), sore throat (23.5%), and runny nose (21.9%) being most common. Chronic lung disease (adjusted odds ratio [aOR] 2.36; 95% CI 1.42–3.92), presence of ≥1 chronic condition (aOR 1.58; 95% CI 1.12–2.24), and current smoking (aOR 1.74; 95% CI 1.15–2.63) were independently associated with respiratory symptoms. Influenza vaccination was associated with reduced odds of multi-symptom respiratory illness (aOR 0.72; 95% CI 0.53–0.98). Among symptomatic participants, 29.6% reported antibiotic use, frequently in the absence of febrile illness. Vaccination coverage was high for meningococcal vaccine (96.1%), while pneumococcal vaccination coverage remained lower.

**Conclusion:**

Respiratory symptoms remain a substantial health burden among Hajj pilgrims across consecutive seasons. Chronic lung disease and smoking increase susceptibility, while influenza vaccination appears protective. The frequent use of antibiotics highlights the need for strengthened antimicrobial stewardship. Enhanced vaccination uptake, targeted risk communication, and integrated prevention strategies are critical to mitigating respiratory infection risks during mass gatherings and safeguarding global public health.

## Introduction

Mass gatherings pose unique and complex public health challenges, particularly with regard to the transmission of respiratory infections. The World Health Organization (WHO) defines a mass gathering as an event that attracts a sufficient number of people to strain the planning and response resources of the host community. Such events facilitate close interpersonal contact, crowd density, shared accommodations, and international travel—all of which increase the risk of rapid spread of respiratory pathogens (1).

The annual Hajj pilgrimage to Makkah in Saudi Arabia represents one of the largest recurring mass gatherings globally, drawing millions of pilgrims from over 180 countries each year. The demographic diversity of pilgrims—including older adult individuals and those with chronic comorbidities—combined with intense crowding during rituals, creates an environment highly conducive to respiratory disease transmission (2, 3). Previous studies have consistently identified respiratory tract infections as the most frequently reported health problem among Hajj pilgrims, accounting for a substantial proportion of healthcare visits during and after the pilgrimage (4). Recent evidence from post-COVID-19 mass gatherings, including Hajj, indicates that respiratory tract infections remain a major health burden among pilgrims despite strengthened preventive measures such as expanded vaccination campaigns, enhanced surveillance systems, and infection prevention strategies. Studies conducted in recent pilgrimage seasons have consistently shown that influenza-like illness remains highly prevalent, with respiratory viruses such as influenza, rhinovirus, and seasonal coronaviruses continuing to dominate the etiological profile of respiratory infections among pilgrims (5–8).

In addition, post-pandemic surveillance data from Hajj and similar mass gatherings demonstrate that close contact, crowding, and international mobility continue to facilitate respiratory pathogen transmission, reinforcing the persistent vulnerability of pilgrims to acute respiratory infections (6, 9).

Recent studies have also raised concerns regarding antimicrobial use during Hajj, reporting frequent empirical antibiotic consumption for predominantly viral respiratory presentations, thereby contributing to ongoing risks of antimicrobial resistance dissemination in global mass gathering settings (5, 10, 11).

Respiratory infections during Hajj range from mild upper respiratory tract infections to severe illnesses such as influenza, pneumonia, and, historically, Middle East respiratory syndrome coronavirus (MERS-CoV) (12). Viral pathogens, particularly influenza viruses, rhinoviruses, and seasonal coronaviruses, are commonly detected, although bacterial pathogens including *Streptococcus pneumoniae* and *Neisseria meningitidis* also contribute to disease burden (13–15). The global mobility of pilgrims further raises concerns about cross-border dissemination of infectious agents following the pilgrimage (16).

Vaccination remains a cornerstone of prevention strategies for respiratory infections in mass gatherings. Meningococcal vaccination is mandatory for Hajj attendance, while influenza and COVID-19 vaccines are strongly recommended, especially for high-risk groups such as older adults and individuals with chronic diseases (9). Pneumococcal vaccination is also advised for specific risk categories. However, despite these recommendations, vaccine uptake varies by country of origin, access, awareness, and risk perception (17).

In addition to vaccination status, several individual-level factors may influence susceptibility and clinical outcomes, including age, underlying medical conditions (e.g., diabetes, chronic lung disease, cardiovascular disease), smoking behavior, and recent travel history (18). Furthermore, inappropriate or empirical antibiotic use for predominantly viral respiratory illnesses has been reported among pilgrims, raising concerns about antimicrobial resistance and highlighting the need for improved stewardship interventions in mass gathering settings (11, 19). Recent evidence has further highlighted the complex interaction between chronic respiratory diseases such as chronic obstructive pulmonary disease (COPD) and cardiovascular comorbidities, particularly in relation to respiratory infection severity and outcomes. COPD has also been shown to significantly increase susceptibility to community-acquired pneumonia, especially among patients receiving inhaled corticosteroid therapy, which may modify infection risk profiles and disease burden (20, 21). Moreover, environmental exposures such as air pollution (smog) and extreme heat have been increasingly associated with worsened respiratory and cardiovascular outcomes, while socioeconomic disparities further modify these risks in vulnerable populations (22, 23). Additionally, recurrent respiratory infections have been linked to underlying immunodeficiency indicators, emphasizing the importance of host susceptibility factors in respiratory disease epidemiology (24, 25).

Although numerous studies have examined respiratory illnesses during single Hajj seasons, there remains limited multi-season comparative data integrating clinical symptoms, vaccination history, comorbidities, smoking exposure, and antibiotic consumption within the same analytical framework. Understanding temporal trends and determinants across consecutive pilgrimage seasons is critical for refining preventive policies, strengthening risk communication, and enhancing preparedness strategies. Although vaccination campaigns for influenza and COVID-19 have expanded following the pandemic, recent studies indicate persistent heterogeneity in vaccine uptake among pilgrims, particularly among younger and low-risk individuals, with suboptimal coverage still reported in several cohorts (26, 27).

Recent analyses of antimicrobial use during Hajj continue to demonstrate high rates of non-prescribed or empirical antibiotic consumption for respiratory symptoms, reinforcing concerns regarding inappropriate use and the potential contribution of mass gatherings to the global spread of antimicrobial resistance (28, 29).

Despite the growing body of literature on respiratory infections during Hajj, existing studies remain largely fragmented, often focusing on isolated seasons, single outcomes, or limited risk factor sets. Integrated evidence combining respiratory symptoms, vaccination uptake, comorbidities, smoking behavior, and antibiotic consumption across multiple consecutive Hajj seasons is still lacking. This limits the ability to assess temporal stability or change in risk patterns in response to evolving public health interventions.

Therefore, this study aims to assess the burden and determinants of respiratory infections among pilgrims during three consecutive Hajj seasons (2023–2025). Specifically, it seeks to examine the prevalence of respiratory symptoms, vaccination coverage, comorbidities, smoking behaviors, antibiotic use patterns, and associated risk factors, with the goal of generating evidence to inform targeted public health interventions and strengthen preventive strategies for future mass gatherings.

## Methods

### Study design and setting

This study was a multi-season cross-sectional investigation conducted during three consecutive Hajj seasons (2023, 2024, and 2025) in Makkah, Saudi Arabia. Hajj is one of the largest annual mass gatherings worldwide, attracting millions of pilgrims from diverse geographic, cultural, and epidemiological backgrounds. Data collection was carried out during the peak Hajj period each year in collaboration with field teams stationed at designated pilgrim accommodations and healthcare access points.

The study aimed to assess the burden and determinants of respiratory infections among pilgrims and to evaluate associations with vaccination status, comorbidities, smoking behavior, and antibiotic use.

### Study population and sampling

Eligible participants were adult pilgrims (≥18 years) attending Hajj during the study years who were willing to participate in the study and provided informed consent. Participants were recruited regardless of nationality. The questionnaire was administered in Arabic or English, depending on participant preference and interviewer availability.

Both male and female pilgrims of all nationalities were eligible for inclusion. Individuals who declined participation or were unable to provide informed consent were excluded.

A convenience sampling strategy was employed due to operational constraints inherent to the Hajj environment. Trained data collectors approached pilgrims in organized Hajj groups and healthcare-access settings. Over the three seasons, approximately 700 pilgrims were enrolled.

Each participant was assigned a unique sample barcode to ensure accurate linkage between questionnaire data and collected respiratory samples (where applicable) while maintaining anonymity.

Due to the operational constraints of the Hajj environment, participants were recruited using a convenience sampling approach, primarily from organized pilgrim groups and healthcare-access points. This may have introduced selection bias, potentially overrepresenting individuals with higher healthcare contact or health-seeking behavior.

### Data collection procedures

Data were collected using a standardized structured questionnaire that was harmonized across the three seasons, with minor refinements introduced to improve clarity and completeness. The questionnaire was administered by trained data collectors in the participant’s preferred language when feasible.

The instrument captured the following domains:

1. Sociodemographic characteristics

Age, gender, nationality, type of Hajj attendee (e.g., pilgrim category or group affiliation), and recent travel history (within six months prior to Hajj).

Vaccination status

Self-reported receipt of: Meningococcal vaccine (within the past five years), Influenza vaccine (current season), COVID-19 vaccine (any dose), and Pneumococcal vaccine (within the past ten years).

Participants who had not received recommended vaccines were asked to specify reasons where applicable.

Medical history and comorbidities

Self-reported chronic conditions including: Cardiovascular disease, Hypertension, Chronic lung diseases (e.g., asthma, COPD, bronchiectasis), Diabetes mellitus (Type 1 or Type 2), Chronic kidney disease, Chronic neurological disorders, Immune suppression (e.g., HIV infection, malignancy, prolonged immunosuppressive therapy), other self-reported chronic illnesses.

Smoking and exposure history

Current and past use of cigarettes, hookah/shisha, pipes/cigars, and electronic cigarettes. Duration (years) and frequency (per day/week) were recorded where available.

Respiratory and systemic symptoms

Participants were asked whether they were currently experiencing any of the following symptoms at the time of interview: Feeling feverish, Runny nose, Cough, Sore throat, Shortness of breath, Headache, Muscle pain, Sputum/phlegm, Lethargy/tiredness, Shivering, Gastrointestinal symptoms (diarrhea, vomiting).

For each reported symptom, duration in days was documented. Participants were also asked whether they had experienced a resolved episode of respiratory illness since arrival in Makkah, whether they had sought medical care, and whether they had self-medicated.

1. Antibiotic use

Participants reporting respiratory symptoms were asked whether they had taken antibiotics. If yes, information was collected regarding the name of the antibiotic (if known), timing of initiation and cessation, and dosage where available.

### Case definition

For analytical purposes, a respiratory infection case was defined as the presence of one or more acute respiratory symptoms (e.g., cough, sore throat, runny nose, shortness of breath) with or without systemic features such as fever or myalgia at the time of assessment. Sensitivity analyses were planned using stricter definitions, such as the presence of at least two respiratory symptoms or respiratory symptoms plus fever.

### Statistical analysis

Descriptive statistics were used to summarize participant characteristics across the three Hajj seasons (2023–2025). Categorical variables were presented as frequencies and percentages. Continuous variables were summarized using means and standard deviations or medians and interquartile ranges, depending on distribution. Comparisons between seasons (2023–2025) were performed using chi-square or Fisher’s exact tests for categorical variables and t-tests or non-parametric equivalents for continuous variables.

To identify factors associated with respiratory infection, univariable analyses were first conducted. Variables with *p* ≤ 0.200 in univariable analysis and those considered clinically relevant were entered into multivariable logistic regression models. Adjusted odds ratios (aORs) with 95% confidence intervals (CIs) were reported.

Separate models were constructed to examine:

- Determinants of respiratory symptoms.
- Predictors of antibiotic use.
- Associations between vaccination status and symptom occurrence

Statistical significance was defined as a two-sided *p*-value ≤ 0.050. Variables with *p* ≤ 0.200 in univariable analysis were included in the multivariable model.

## Results

### Participant characteristics

A total of 712 human participants (adult Hajj pilgrims) were enrolled across three consecutive Hajj seasons: 228 in 2023, 241 in 2024, and 243 in 2025. The mean age of participants was 52.6 years (SD ± 14.8), with 29.4% aged ≥60 years. Males constituted 58.7% of the sample. Pilgrims represented more than 25 nationalities, predominantly from Asia (46.2%) and Africa (32.8%), followed by the Middle East and Europe.

Overall, 41.3% of participants reported at least one chronic medical condition. The most frequently reported comorbidities were hypertension (24.7%), diabetes mellitus (19.5%), and chronic lung disease (8.6%). Current smoking (any form) was reported by 18.9%, while 9.7% were ex-smokers.

Vaccination coverage varied by vaccine type. Meningococcal vaccination coverage was 96.1%, COVID-19 vaccination 81.7%, and pneumococcal vaccination 37.5%. Uptake of recommended vaccines was significantly higher among pilgrims aged ≥60 years (*p* < 0.001) (Table 1).

**Table 1:** Sociodemographic and clinical characteristics of participants (*N* = 712).

Characteristic	*n* (%)
Gender	
Male	418 (58.7)
Female	294 (41.3)
Age group (years)	
<40	176 (24.7)
40–59	327 (45.9)
≥60	209 (29.4)
≥1 Chronic condition	294 (41.3)
Hypertension	176 (24.7)
Diabetes mellitus	139 (19.5)
Chronic lung disease	61 (8.6)
Heart disease	54 (7.6)
Immune suppression	22 (3.1)
Current smoker (any type)	135 (18.9)
Vaccination status	
Meningococcal	684 (96.1)
COVID-19 (any dose)	582 (81.7)
Pneumococcal	267 (37.5)

### Prevalence of respiratory symptoms

At the time of assessment, 38.9% (n = 277) of pilgrims reported at least one respiratory symptom. When applying a stricter definition (≥2 respiratory symptoms or respiratory symptoms with fever), the prevalence was 24.6%.

The most frequently reported symptoms were cough (28.1%), sore throat (23.5%), runny nose (21.9%), and feeling feverish (14.3%). Shortness of breath was reported by 9.8%, more commonly among participants aged ≥60 years and those with chronic lung disease (*p* < 0.01). The median symptom duration was 3 days (IQR 2–5). Participants were additionally asked to specify whether their current respiratory symptoms began before arrival in Makkah or developed after arrival during the Hajj period. This information was used to contextualize symptom onset; however, detailed etiological classification (imported versus Hajj-acquired infection) was not possible due to reliance on self-reported timing.

Symptom prevalence was highest in 2024 (42.7%), followed by 2025 (39.1%) and 2023 (34.6%), though the difference did not reach statistical significance (*p* = 0.080) (Table 2). Regarding symptom onset, a proportion of participants reported that their symptoms began after arrival in Makkah, while others indicated symptom onset prior to arrival; however, detailed stratification of imported versus locally acquired infections was not performed.

**Table 2:** Prevalence of reported symptoms among participants.

Symptom	*n* (%)
≥1 Respiratory symptom	277 (38.9)
Cough	200 (28.1)
Sore throat	167 (23.5)
Runny nose	156 (21.9)
Feeling feverish	102 (14.3)
Headache	121 (17.0)
Muscle pain	98 (13.8)
Shortness of breath	70 (9.8)
Sputum/phlegm	113 (15.9)
Lethargy/tiredness	145 (20.4)

### Healthcare seeking and antibiotic use

Among symptomatic participants (*n* = 277), 32.9% visited a healthcare facility, 46.2% reported self-medication, and 29.6% reported antibiotic use during their illness episode.

Antibiotic use was significantly more common among individuals reporting fever (aOR 2.14; 95% CI 1.31–3.49) and among those who sought healthcare (aOR 3.87; 95% CI 2.29–6.54). Notably, 61.8% of antibiotic users did not report fever, suggesting potential empirical use in cases likely to be viral respiratory infections.

Where antibiotic class information was available, the most frequently reported agents included macrolides and beta-lactam antibiotics; however, detailed classification was limited by incomplete recall among participants. This limitation reflects the challenges of self-reported antimicrobial data collection in field-based mass gathering settings.

### Vaccination status and respiratory symptoms

Influenza vaccination was associated with a lower likelihood of reporting ≥2 respiratory symptoms (aOR 0.68; 95% CI 0.49–0.94). Pneumococcal vaccination showed a protective trend among participant’s ≥ 60 years but did not reach statistical significance in the full model.

COVID-19 vaccination status was not significantly associated with overall respiratory symptom reporting, though vaccinated individuals had lower odds of reporting feverish illness (*p* = 0.040).

### Risk factors for respiratory symptoms

In multivariable logistic regression analysis, independent predictors of reporting ≥1 respiratory symptom included: Chronic lung disease (aOR 2.36; 95% CI 1.42–3.92), Current smoking (aOR 1.74; 95% CI 1.15–2.63), and Presence of ≥1 chronic condition (aOR 1.58; 95% CI 1.12–2.24).

Influenza vaccination was independently associated with reduced odds of respiratory symptoms (aOR 0.72; 95% CI 0.53–0.98) (Table 3).

**Table 3:** Multivariable logistic regression for ≥1 respiratory symptom.

Variable	Adjusted OR	95% CI	*p*-value
Age ≥60 years	1.18	0.83–1.69	0.350
≥1 Chronic condition	1.58	1.12–2.24	0.009
Chronic lung disease	2.36	1.42–3.92	0.001
Current smoker	1.74	1.15–2.63	0.008
Influenza vaccinated	0.72	0.53–0.98	0.037
COVID-19 vaccinated	0.91	0.63–1.31	0.620

### Seasonal trends

While overall vaccination coverage remained stable across the three seasons, uptake of recommended respiratory vaccines increased modestly across the three seasons. Antibiotic use among symptomatic individuals decreased slightly over time (32.4% in 2023 vs. 26.1% in 2025), though the trend was not statistically significant (*p* = 0.180).

## Discussion

In this multi-season study of pilgrims attending Hajj between 2023 and 2025 in Makkah, Saudi Arabia, respiratory symptoms emerged as a substantial and persistent public health concern, affecting nearly two-fifths of participants. Chronic lung disease, smoking, and the presence of underlying comorbidities were independently associated with increased odds of respiratory illness, whereas influenza vaccination was associated with a reduced risk of respiratory symptoms.

In addition, antibiotic use was common among symptomatic pilgrims, including among those without clear febrile illness, raising concerns regarding antimicrobial stewardship in mass gathering settings.

Respiratory tract infections have historically been the most frequently reported health problem during Hajj, accounting for a large proportion of outpatient visits and self-reported illness (3, 4, 30).

Our findings are consistent with prior investigations demonstrating that cough, sore throat, and rhinorrhea are the most prevalent symptoms during pilgrimage seasons (11, 13). The high density of pilgrims, shared accommodations, physically demanding rituals, and prolonged close contact facilitate person-to-person transmission of respiratory pathogens.

Mass gatherings such as Hajj are recognized by the World Health Organization as environments that amplify communicable disease transmission due to crowding, international travel, and strain on health systems (1). The recurring nature of respiratory illness across three consecutive seasons in our study reinforces the need for sustained, rather than seasonal, preparedness strategies.

Our analysis identified chronic lung disease and smoking as strong predictors of respiratory symptoms. These findings align with broader epidemiological evidence indicating that pre-existing pulmonary conditions and tobacco exposure increase susceptibility to respiratory infections and worsen symptom severity (31, 32). Given that nearly one-fifth of our participants were current smokers, targeted pre-Hajj risk communication may be warranted. Recent literature has also emphasized that chronic respiratory diseases such as COPD often coexist with cardiovascular disease, creating a synergistic effect that increases vulnerability to respiratory infections and adverse outcomes. In addition, inhaled corticosteroid use in COPD patients has been associated with increased risk of pneumonia in community settings, which may partially explain increased susceptibility observed in high-risk populations (33–35). Environmental exposures, including air pollution and heat stress, as well as underlying immunodeficiency states, have also been identified as important modifiers of respiratory infection risk in recent studies (23, 36).

Older age and multimorbidity have also been associated with higher complication rates during Hajj (37). Although age ≥60 years was not independently associated with symptom occurrence in adjusted models, older pilgrims had a higher prevalence of comorbidities and more frequent shortness of breath. This suggests that while exposure risk may be universal, clinical vulnerability remains concentrated among high-risk subgroups.

Vaccination remains a cornerstone of infection prevention strategies during Hajj. In our cohort, meningococcal vaccination coverage was high, reflecting long-standing entry requirements mandated by Saudi health authorities (10). However, uptake of recommended respiratory vaccines, including influenza and pneumococcal vaccines, remained suboptimal in certain subgroups, particularly among younger pilgrims and those without chronic medical conditions.

Importantly, receipt of seasonal influenza vaccination was independently associated with reduced odds of reporting multiple respiratory symptoms. Although symptom-based outcomes do not confirm laboratory-verified influenza infection, this finding is consistent with previous studies demonstrating reduced influenza-like illness among vaccinated pilgrims (37, 38).

Regarding pneumococcal vaccination, a protective trend was observed but did not reach statistical significance, which may be partly explained by limited statistical power due to the relatively smaller number of vaccinated participants.

Given the intense global mixing of populations during Hajj, strengthening influenza vaccination coverage in countries of origin may contribute to reducing the respiratory illness burden during pilgrimage and potentially limit post-travel transmission.

COVID-19 vaccination coverage in our study was relatively high, reflecting global immunization campaigns following the pandemic. While COVID-19 vaccination was not strongly associated with overall respiratory symptom reporting, vaccinated individuals were less likely to report febrile illness. This may suggest attenuation of disease severity rather than complete prevention of infection, consistent with broader post-pandemic observations (39).

Nearly one-third of symptomatic pilgrims reported antibiotic use, and a substantial proportion did so without accompanying fever. Similar patterns have been reported in earlier Hajj studies, where antibiotics were frequently used empirically for predominantly viral respiratory illnesses (11, 15). This pattern raises important concerns regarding inappropriate antimicrobial use and the potential contribution to antimicrobial resistance (AMR).

Where antibiotic class information was available, commonly reported agents included macrolides (e.g., azithromycin) and beta-lactam antibiotics (including amoxicillin-based regimens). However, detailed classification was limited by incomplete participant recall, reflecting the challenges of collecting precise antimicrobial data in field-based mass gathering settings.

Mass gatherings provide unique opportunities for the dissemination of resistant organisms across international borders (40, 41). Unregulated or self-directed antibiotic consumption during pilgrimage may further amplify this risk. Strengthening antimicrobial stewardship messaging, improving clinical triage, and enhancing awareness of the distinction between viral and bacterial respiratory illness could help reduce unnecessary antibiotic exposure in such settings.

The persistence of respiratory illness across multiple seasons underscores the importance of integrated prevention strategies. These include:

1. Enhancing pre-travel vaccination campaigns, particularly for recommended respiratory vaccines among high-risk groups.
2. Promoting non-pharmaceutical interventions such as mask use in crowded settings, hand hygiene, and respiratory etiquette.
3. Targeted education for smokers and individuals with chronic lung disease.
4. Reinforcing antimicrobial stewardship principles among healthcare providers and pilgrims.

The Ministry of Health in Saudi Arabia has implemented comprehensive public health measures during Hajj, including vaccination requirements and surveillance systems. Our findings support continued investment in these efforts and suggest that more tailored risk communication strategies may further reduce respiratory disease burden.

This study has several strengths. It spans three consecutive Hajj seasons, allowing for assessment of temporal consistency. It integrates detailed data on vaccination, comorbidities, smoking exposure, symptom duration, and antibiotic use within a unified analytical framework. The relatively large sample size enhances statistical power for multivariable analysis.

However, limitations should be acknowledged. First, Self-reported symptom data may be subject to recall bias and misclassification. In addition, the use of convenience sampling, with recruitment primarily conducted in organized pilgrim accommodations and healthcare-access points, may have introduced selection bias. This could have led to an overrepresentation of pilgrims with higher healthcare utilization or greater health awareness, potentially limiting the generalizability of the findings to the wider pilgrim population. Second, the cross-sectional design limits causal inference. Third, convenience sampling may affect generalizability, although participant diversity by nationality and age increases external validity. Fourth, although symptom onset timing was recorded, the study was not designed to distinguish between imported and Hajj-acquired respiratory infections, limiting etiological interpretation of infection origin. Finally, the absence of systematic laboratory confirmation for all cases limited the ability to determine pathogen-specific etiologies.

## Conclusion

Respiratory symptoms remain a substantial health burden among Hajj pilgrims across consecutive seasons. Chronic lung disease, smoking, and multimorbidity increase susceptibility, while vaccination appears protective against respiratory illness. The frequent use of antibiotics, including in the absence of clear bacterial indicators, highlights ongoing stewardship challenges. As one of the world’s largest recurring mass gatherings, Hajj offers both a challenge and an opportunity for global health. Strengthening vaccination uptake, targeted risk communication, and antimicrobial stewardship interventions will be essential to mitigate respiratory infection risks and safeguard both local and global public health.

## Data Availability

The raw data supporting the conclusions of this article will be made available by the authors, without undue reservation.
